# Exploring the Additive Benefit of PTSD Treatment on Eating Disorder Outcomes for Those with Co-Occurring PTSD

**DOI:** 10.3390/bs15091173

**Published:** 2025-08-29

**Authors:** Christina R. Felonis, Alexandra D. Convertino, Erin E. Reilly, Sanvi Beri, Kimberly Claudat

**Affiliations:** 1 Eating Disorders Center for Treatment and Research, Department of Psychiatry, University of California, San Diego, CA 92121, USA; sberi@health.ucsd.edu (S.B.); kclaudat@health.ucsd.edu (K.C.); 2 Department of Psychology, California State University, Dominguez Hills, CA 90747, USA; aconvertino@csudh.edu; 3 Eating Disorders Program, Department of Psychiatry and Behavioral Sciences, University of California, San Francisco, CA 94107, USA; erin.reilly3@ucsf.edu

**Keywords:** eating disorders, posttraumatic stress disorder, co-occurring disorders, trauma, integrated treatment, treatment outcomes, psychotherapy, body dissatisfaction, moderation

## Abstract

In the current study, we conducted a preliminary test of whether adding evidence-based PTSD treatment may influence intensive ED treatment outcomes by comparing individuals with co-occurring ED-PTSD diagnoses who received PTSD treatment to those who did not. We hypothesized that receiving PTSD treatment would moderate outcomes, such that individuals who received PTSD treatment throughout their course of ED treatment would demonstrate improved outcomes compared to those with PTSD who did not. The participants were 104 adults with *DSM-5* EDs and PTSD admitted to a partial-hospital ED program. The participants either completed a course of evidence-based PTSD treatment within the context of intensive ED treatment or completed ED treatment as usual (*n* = 58 PTSD treatment, *n* = 46 TAU). Multilevel modeling was used to examine changes in symptoms as moderated by receipt of PTSD treatment over the course of treatment and at follow-up. Models exploring changes in anxiety, depression, clinical impairment, and ED symptoms suggested no differences across groups. The findings indicated that individuals who received PTSD treatment began with higher body dissatisfaction compared to their peers and experienced a significant decrease over the course of treatment. This investigation provides preliminary support for improved body dissatisfaction outcomes when PTSD treatment is incorporated into intensive ED treatment.

## 1. Introduction

Eating disorders (EDs), including anorexia nervosa (AN), bulimia nervosa (BN), and binge eating disorder, are psychiatric conditions associated with significant physical, psychological, and functional impairment (Johnson et al., 2001; Tan et al., 2023). Increasingly, research has highlighted the co-occurrence of EDs with posttraumatic stress disorder (PTSD). Evidence suggests that trauma exposure is common among individuals with EDs in community settings and that approximately 25–40% of those with EDs meet the diagnostic criteria for PTSD or report clinically significant trauma symptoms (Ferrell et al., 2022; Mitchell et al., 2021). Prevalence rates are even higher within intensive levels of care for EDs where estimates indicate that up to 50% of individuals present with co-occurring PTSD or substantial PTSD symptomatology (Gleaves et al., 1998; Vanzhula et al., 2019).

The co-occurrence of EDs and PTSD (ED-PTSD) has led researchers to explore the nature of this relationship, with a growing body of evidence suggesting a functional and bidirectional association between the disorders. Individuals with PTSD may utilize ED behaviors such as dietary restriction, binge eating, and purging as maladaptive strategies to cope with and/or avoid trauma-related intrusions, negative thoughts and emotions, and hyperarousal (Mitchell et al., 2021; Trottier et al., 2016). These behaviors can serve as temporary forms of regulation, numbing, or distraction in the face of overwhelming internal experiences, thereby maintaining both disorders (Mitchell et al., 2021; Trottier et al., 2016). Additionally, the psychological features associated with EDs such as negative self-criticism, low distress tolerance, and preoccupation with weight/shape may intensify PTSD symptomatology, hinder trauma processing, and perpetuate trauma responses (Vanzhula et al., 2019). This reciprocal and reinforcing dynamic may then contribute to poor treatment outcomes for ED-PTSD. Individuals with both conditions tend to present with more complex clinical profiles, including heightened suicidality, emotion dysregulation, and ED symptomatology at the start of treatment (Brewerton, 2019; Mitchell et al., 2021). These individuals are at elevated risk for premature treatment dropout, suboptimal response to ED interventions, and relapse following initial symptom remission (Convertino & Mendoza, 2023). As a result, there has been growing interest in adapting or integrating trauma-focused therapies within the context of ED treatment, particularly at higher levels of care where symptom acuity is greatest (Brewerton, 2023).

Several promising treatment approaches for ED-PTSD have emerged in recent years. Two evidence-based treatments for PTSD, prolonged exposure (PE) and cognitive processing therapy (CPT), have been implemented in a partial hospitalization program (PHP) for EDs, with preliminary results demonstrating feasibility and significant reductions across ED and PTSD symptoms (Claudat et al., 2022). The current study sought to build on the findings of Claudat et al. (2022), which can be referenced for a more in-depth review of the existing literature on integrated ED-PTSD treatment. Furthermore, a manualized protocol combining CPT with cognitive behavioral therapy (CBT) for EDs has been developed and evaluated in open and randomized controlled trials, with findings indicating that the integrated intervention is acceptable and effective in reducing PTSD symptoms following intensive ED treatment (Trottier et al., 2022; Trottier et al., 2017).

While these integrated approaches demonstrate clinical potential for reducing ED and PTSD symptomatology, it remains unclear whether individuals with ED-PTSD who receive integrated care experience greater improvements in symptoms than those who receive standard ED care alone. The question therefore remains whether trauma-focused interventions not only alleviate PTSD symptoms but also contribute meaningfully to ED recovery above and beyond treatment as usual (TAU). Given the theoretical framework of the functional and bidirectional association between these disorders, it is possible that incorporating trauma-focused interventions into ED treatment may simultaneously target important maintenance factors for EDs. For example, reducing trauma-related distress and the need to engage in ED behaviors to maladaptively cope with this distress could have a unique impact on ED symptomatology that has yet to be conclusively established. Similarly, given that individuals with ED-PTSD are at elevated risk for suboptimal ED treatment outcomes, simultaneously reducing trauma-related distress may enhance their capacity to engage successfully in ED treatment. However, given the substantial investment of time, training, and institutional resources required to implement integrated protocols, it is imperative to first evaluate whether targeting PTSD in ED populations yields additive benefits for EDs over TAU alone. The current study sought to address this gap in the existing literature by comparing outcomes among adults with ED-PTSD who did and did not receive PTSD treatment during a PHP for EDs. It was specifically hypothesized that concurrent ED and PTSD treatment would moderate outcomes, such that individuals who received integrated care would demonstrate improved outcomes compared to those who did not, defined as greater reductions in eating pathology, depression, anxiety, and clinical impairment.

## 2. Materials and Methods

### 2.1. Participants and Procedures

The participants were 104 adult patients admitted to a PHP for EDs between October 2016 and November 2023. The participants met the diagnostic criteria for PTSD and the following EDs: AN-restricting type (*n* = 28), AN-binge/purge type (*n* = 20), BN (*n* = 25), avoidant/restrictive food intake disorder (*n* = 3), and other specified feeding or eating disorder (*n* = 28). In cases of multiple admissions (*n* = 4), only data from the first admission where the patients received trauma treatment were analyzed. To assess ED and PTSD diagnoses, the participants completed structured clinical interviews at intake with either the MINI International Neuropsychiatric Interview (Sheehan et al., 1998) or the Structured Clinical Interview for DSM-5 (First et al., 2015) administered by trained bachelor’s-level research assistants under the supervision of licensed clinical psychologists. The participants also completed self-report measures at admission, 1 month into treatment, discharge, 6-month follow-up, and 1-year follow-up. All study procedures were approved by the University of California, San Diego Institutional Review Board.

#### 2.1.1. Treatment as Usual

Treatment in the PHP included 6–10 hours of daily programming, including individual, family, and group treatment. The program is primarily based on full-model dialectical behavior therapy (DBT), including weekly skills groups, phone coaching, individual therapy, and consultation teams. Additional therapeutic modalities such as Acceptance and Commitment Therapy, CBT, and Radically Open DBT were also incorporated. Treatment teams were multidisciplinary, including psychology, social work, psychiatry, nursing, and nutrition professionals. See Brown et al. (2018) for further details about programming.

#### 2.1.2. PTSD Treatment

Trauma treatment options included 12 sessions of CPT (Resick et al., 2017) and 9–12 sessions of PE (Foa et al., 2019), depending on symptom improvement and individual progress made. Treatment protocols were selected based on clinician expertise and patient preference. All patients diagnosed with PTSD at intake were considered for trauma treatment. In addition to expressing a desire for trauma treatment, the patients were required to be nutritionally stable (i.e., greater than 85% ideal body weight) and have no imminent safety concerns (e.g., self-harm, suicidality) to receive treatment. These eligibility criteria were based on prior eligibility criteria for DBT-PE (Harned et al., 2012) and CPT for EDs (Trottier et al., 2022). Due to clinical procedures that evolved over time, information on whether trauma protocols were successfully completed was only available for 56 patients (approximately 50% of the sample). Of these patients, 43 completed trauma treatment and 13 did not due to various reasons, including premature discharge, transfer to a residential treatment center, increase in treatment-interfering behaviors, and patient desire to pause or discontinue.

### 2.2. Measures

#### 2.2.1. Eating Disorder Symptoms

ED symptoms were measured with the Eating Disorder Examination Questionnaire (EDE-Q) (Fairburn & Beglin, 1994) for all patients. The Eating Pathology Symptoms Inventory (EPSI) (Forbush et al., 2013) was added to the assessment protocol later and was therefore only administered to a subset of patients (*n* = 93). For the EDE-Q, items were scored on a 7-point scale ranging from 0 (no days) to 6 (every day), with higher scores indicating greater eating pathology. The measure produces four subscales (Dietary Restraint, Shape Concern, Weight Concern, and Eating Concern), as well as a Global score. In the current study, Cronbach’s alpha ranged from 0.95 to 0.97 for the Global score, 0.84 to 0.91 for the Dietary Restraint subscale, 0.92 to 0.97 for the Shape Concern subscale, 0.83 to 0.91 for the Weight Concern subscale, and 0.68 to 0.82 for the Eating Concern subscale across timepoints. For the EPSI, items were scored on a 5-point scale ranging from 0 (never) to 4 (very often), with higher scores indicating greater eating pathology. It produces eight subscales (Body Dissatisfaction, Binge Eating, Cognitive Restraint, Purging, Restricting, Excessive Exercise, and Muscle Building; Negative Attitudes towards Obesity was omitted from the current study due to infrequent use). In the current study, Cronbach’s alpha ranged from 0.85 to 0.93 for the Body Dissatisfaction subscale, 0.83 to 0.82 for the Binge Eating subscale, 0.74 to 0.92 for the Cognitive Restraint subscale, 0.70 to 0.81 for the Purging subscale, 0.86 to 0.92 for the Restricting subscale, 0.85 to 0.93 for the Excessive Exercise subscale, and 0.23 to 0.71 for the Muscle Building subscale across timepoints. Given the low internal consistency for the Muscle Building subscale, analyses with this subscale are not reported. Previous studies have similarly reported low internal consistency in ED samples (Forbush et al., 2013), perhaps indicating that the items on this scale are not a unitary construct within clinical samples.

#### 2.2.2. Anxiety Symptoms

Anxiety symptoms were measured by the Trait subscale of the State-Trait Anxiety Inventory (Speilberger et al., 1983). Items were scored on a 4-point scale ranging from 1 (not at all) to 4 (very much so), with higher scores indicating greater anxiety. In the current study, Cronbach’s alpha ranged from 0.86 to 0.95 across timepoints.

#### 2.2.3. Depression Symptoms

Depression symptoms were measured using the Patient Health Questionnaire-9 (Kroenke & Spitzer, 2002). Items were scored on a 4-point scale ranging from 0 (not at all) to 3 (nearly every day), with higher scores indicating greater depression. Given changes in assessment protocols over time, this measure was administered to a subset of patients (*n* = 68). In the current study, Cronbach’s alpha ranged from 0.81 to 0.91 across timepoints.

#### 2.2.4. Eating Disorder-Related Clinical Impairment

ED-related clinical impairment was measured with the Clinical Impairment Assessment Questionnaire (Bohn & Fairburn, 2008). Items were scored on a 5-point scale ranging from 0 (not at all) to 4 (a lot), with higher scores indicating greater impairment. Given changes in assessment protocols over time, this measure was administered to a subset of patients (*n* = 93). In the current study, Cronbach’s alpha ranged from 0.93 to 0.97 across timepoints.

### 2.3. Analysis

All analyses were performed using R Statistical Software (R Core Team, 2025; version 4.5.0), a statistical programming language capable of handling multilevel data structures. R was chosen for its extensive library of open-source packages, support for reproducible workflows, and flexibility in customizing analyses. Linear multilevel modeling analyses were conducted using the “lme4” package (Bates et al., 2015) with full information maximum likelihood estimation to account for missing data. The models included the fixed effects of time, trauma treatment receipt, and the interaction thereof to examine moderation effects, as well as random effects for patient and time. Given the preliminary and naturalistic nature of the current study, an a priori power analysis was not conducted and corrections were not made for multiple comparisons. Some researchers recommend post hoc power analyses, given that power analyses in multilevel modeling vary substantially based on the input parameters (Arend & Schäfer, 2019). Following the procedures outlined in Arend and Schäfer (2019), post hoc power analyses were conducted using Monte Carlo simulation within the “simr” package in R (Green & MacLeod, 2016). Based on 1000 simulations per model, the estimated power to detect the fixed effect of time ranged from 0.90 to 1.00, trauma treatment receipt ranged from 0.24 to 0.53, and the interaction ranged from 0.71 to 0.98, given the unique variance structure in each model and assuming a medium standardized effect size (Cohen’s *d* = 0.5) for all fixed effects. The current study therefore had sufficient power to detect group-by-time interactions, but was underpowered for detecting overall differences between groups. Interpretation of the nonsignificant main effects of trauma treatment receipt should therefore be made with caution. See the Supplementary Materials for full results, including 95% confidence intervals.

## 3. Results

Demographic information for the current sample is reported in Table 1. In brief, the patients were mostly women (*n =* 90; 86.5%), white (*n* = 91; 87.5%), and non-Hispanic/Latinx (*n* = 84; 80.8%). At admission, the average age was 27.9 years (SD = 10.8). Of the 104 patients, 58 (55.8%) received trauma treatment, with 34 receiving CPT and 24 receiving PE. There were significant differences between the patient group that received trauma treatment and the group that did not. Patients who received trauma treatment had a younger age of ED onset, higher BMI at admission, and higher BMI at discharge than patients who did not receive trauma treatment. No other significant differences were observed (see Table 1).

**Figure 1:**
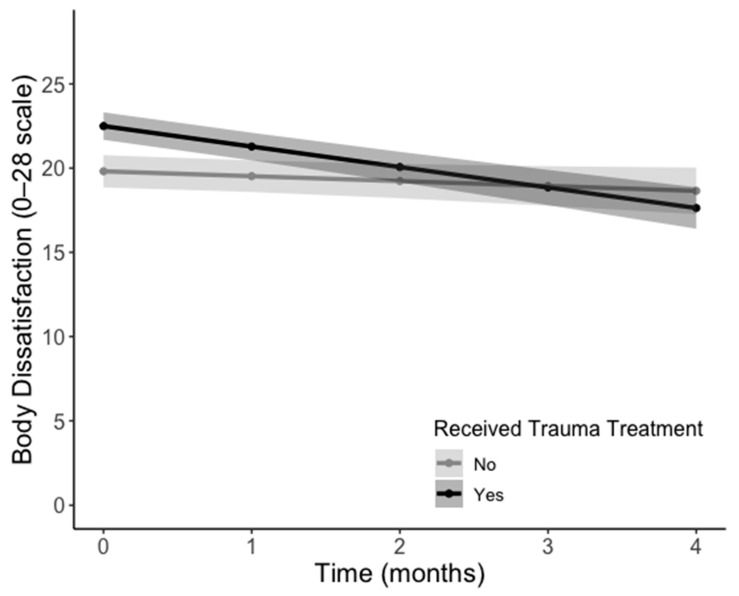
Change in body dissatisfaction over time by trauma treatment status (*p* = 0.025). Lines represent estimated marginal means from multilevel models; shaded areas represent 95% confidence intervals.

All models were an acceptable fit to the data; see Table 2 for fit results. There was a significant effect of time in all EDE-Q models such that eating pathology decreased over the course of treatment (*p*s < 0.01). There was no main effect of trauma treatment or the interaction of time and trauma treatment on the EDE-Q subscales. There was a significant main effect of time for the EPSI subscales of Cognitive Restraint (*β* = −0.44, *SE* = 0.17, *t*(81.82) = −5.58, *p* = 0.012) and Restricting (*β* = −1.11, *SE* = 0.35, *t*(75.52) = −3.15, *p* = 0.002), indicating a decrease over the course of treatment. Similarly, anxiety (*β* = −2.28, *SE* = 0.57, *t*(88.84) = −4.02, *p* < 0.001), depression (*β* = −0.93, *SE* = 0.42, *t*(54.93) = −2.23, *p* = 0.030), and clinical impairment (*β* = −3.09, *SE* = 0.64, *t*(80.70) = −4.87, *p* < 0.001) also decreased over the course of treatment. There was a main effect of trauma treatment on the EPSI Body Dissatisfaction subscale (*β* = 2.68, *SE* = 1.26, *t*(87.72) = 2.68, *p* = 0.036), but this was qualified by a significant interaction effect between trauma treatment and time (*β* = −0.93, *SE* = 0.40, *t*(71.52) = −2.29, *p* = 0.025). Individuals who received trauma treatment began with higher body dissatisfaction compared to their peers and experienced a significant decrease over the course of treatment. Individuals who did not receive trauma treatment began with relatively lower body dissatisfaction and did not experience this decrease (see Figure 1). All model effects are presented in Table 3.

## 4. Discussion

The current study contributes to the growing body of literature examining the ED-PTSD intersection within higher levels of care. Patients with ED-PTSD who received integrated care were compared to patients with ED-PTSD who received standard ED treatment alone. The findings suggest that integrating PTSD treatment into a PHP for EDs may not result in additive benefits for eating pathology, depression, anxiety, and clinical impairment outcomes. It appears that patients who received TAU through an intensive, multidisciplinary treatment program for EDs were successful in producing meaningful clinical improvements. However, body dissatisfaction, a well-established and treatment-resistant maintenance factor of ED psychopathology (Glashouwer et al., 2019; Stice & Shaw, 2002), decreased significantly only among patients who received concurrent PTSD treatment, reaching similar levels between the groups by discharge. Notably, these patients started treatment with higher levels of body dissatisfaction and showed meaningful improvements, while those who did not receive trauma treatment started with lower body dissatisfaction and remained relatively stable. This pattern highlights a potential therapeutic pathway through which trauma recovery processes support not only PTSD symptom reduction but also cognitive and emotional shifts key to ED recovery.

The selective impact of integrated treatment on body dissatisfaction may be interpreted through the lens of shared transdiagnostic mechanisms. Individuals with ED-PTSD often present with elevated emotion dysregulation, avoidance, and self-criticism, all of which are implicated in both body image disturbance and trauma-related distress (Mitchell et al., 2021). Trauma-focused interventions, particularly those rooted in exposure-based or cognitive processing frameworks, aim to reduce avoidance, increase distress tolerance, and challenge maladaptive cognitions (Carbajal, 2018). These skills may naturally extend to the domain of body image. For instance, learning to tolerate distressing trauma memories may generalize to tolerating distressing body-related cognitions, reducing the need to engage in ED behaviors, and weakening maladaptive beliefs underlying body dissatisfaction. Similarly, acquiring tools to challenge trauma-based maladaptive beliefs pertaining to self, others, and the world may extend to entrenched ED beliefs pertaining to self and body, creating the opportunity for new learning. It is important to acknowledge that the decrease in body dissatisfaction in patients who received concurrent trauma treatment may also be attributed to their higher body dissatisfaction levels at admission, creating a regression to the mean effect. Additionally, since patients who received concurrent trauma treatment elected to participate and had to meet certain criteria evaluating readiness, it is possible that these individuals were overall more willing to engage in treatment and thus may have benefited more from challenging body dissatisfaction than patients who received standard ED care alone. Further research is needed to support the potential relationship between integrated ED-PTSD treatment and body dissatisfaction and to elucidate the shared transdiagnostic mechanisms underlying it.

These findings offer preliminary support for integrating PTSD treatment into ED care, particularly for individuals with ED-PTSD who present to treatment with elevated levels of body dissatisfaction. Traditionally, ED treatment approaches postpone trauma work due to concerns about destabilizing patients or interfering with nutritional restoration (Brewerton, 2023). However, our results suggest that trauma work may yield change in areas highly resistant to standard ED interventions like body dissatisfaction. If individuals acquire the skills to process traumatic life experiences, sit with discomfort, and shift avoidance patterns, they may be better able to engage in ED-related exposures and challenge longstanding weight/shape-related beliefs. Furthermore, the specific improvement in body dissatisfaction without broader changes in eating pathology, depression, anxiety, and clinical impairment could reflect a “targeted” effect of trauma treatment, producing additive benefits in areas where ED-PTSD pathology intersects most strongly. This suggests that future studies may want to consider incorporating components of trauma-focused interventions into standard treatment for EDs, especially for individuals with treatment-resistant presentations like those with significant body dissatisfaction.

Despite these promising findings, the current study has several limitations. First, the sample was small, comprising predominantly white women, and from a PHP for EDs in the US. The results may therefore not generalize to individuals with ED-PTSD in the broader community, in other contexts, or to men and other gender-diverse individuals who may experience body dissatisfaction and respond to trauma differently. Growing work supports the importance of adapting PTSD treatment to a given individual’s identity and cultural context (Ennis et al., 2020). Therefore, future work is needed to consider whether our findings generalize across different groups and/or whether cultural adaptations of PTSD treatment may enhance outcomes for specific individuals. Second, the multi-component nature of the treatment program, involving various treatment modalities and interdisciplinary care, makes it difficult to attribute the observed clinical changes to PTSD treatment specifically. Third, the study’s naturalistic design prevented standardization of PTSD treatment modality, timing of initiation, duration, and adherence (e.g., whether all patients successfully completed treatment, average number of sessions received), all of which may have influenced the outcomes. For instance, it is possible that patients who received concurrent trauma treatment were those who made more substantial ED progress early on, thereby being appropriate for and having sufficient time to complete a trauma protocol during the PHP. While this may limit the generalizability of our findings to a subset of patients, it could simultaneously offer a source of motivation for effective treatment engagement and behavioral change in patients considering integrated treatment. Fourth, the current study utilized the cognitive and behavioral features of EDs from pre- to post-treatment as the primary outcomes. While the EDE-Q and EPSI are commonly supported as primary outcome measures for ED treatment (Linardon et al., 2017; Rienecke et al., 2022), research utilizing additional markers of progress and longer-term follow-up periods may be valuable in better understanding the impact of integrated care on ED recovery and outcomes. Finally, as indicated by the post hoc power analyses, the current study was adequately powered (assuming 80% power as acceptable) to detect the main effect of time and the interaction effect of time and trauma treatment receipt (with the exception of depression as an outcome). However, all models were underpowered to detect the main effect of treatment. This means that the null effects for trauma treatment receipt observed in the current sample cannot be taken as evidence that there were no significant differences at intake in our selected outcomes for individuals who received trauma treatment compared to those who did not. This leaves the possibility that individuals who received trauma treatment systemically varied based on pre-treatment characteristics from those who did not receive treatment.

To advance this line of inquiry, future studies should aim to test integrated ED-PTSD treatments in randomized controlled trials (RCTs) using large, diverse samples that include a range of diagnoses, trauma types, and care settings. As part of such RCTs, the PTSD treatment protocols would also be standardized, enabling clearer attribution of effects, and consideration of mediators and moderators impacting response to integrated treatment. Additionally, longitudinal designs with follow-up periods would be valuable in determining whether observed changes in body dissatisfaction persist over time and are associated with long-term benefits such as reduced relapse. Finally, neuroscience-informed approaches could help clarify the neurobiological mechanisms underpinning ED-PTSD presentations. Functional imaging or psychophysiological data could reveal whether changes in systems such as emotion regulation circuitry or fear processing contribute to concurrent improvements in body dissatisfaction and PTSD symptoms.

## 5. Conclusions

In the current study, we compared treatment outcomes for two groups of patients with ED-PTSD: those who received PTSD adjunctive treatment and those who did not. Overall, the results indicated that regardless of whether individuals received PTSD adjunctive treatment or not, outcomes were comparable across anxiety, depression, clinical impairment, and eating pathology. However, greater improvements in body dissatisfaction were observed in the group that received concurrent PTSD treatment. Our study provides preliminary evidence that integrating PTSD treatment into intensive ED care may yield additive benefits for body dissatisfaction, a core and persistent feature of ED psychopathology. These findings suggest that trauma-focused interventions may uniquely target the transdiagnostic mechanisms underlying trauma responses and body image disturbance. Rather than viewing trauma work as separate from or disruptive to ED treatment, these results support its role as a complementary and potentially valuable component of care for individuals with ED-PTSD, particularly those with elevated levels of body dissatisfaction.

## Figures and Tables

**Table 1:** Sociodemographic characteristics of the sample by trauma treatment receipt.

Sociodemographic Characteristic		Received Trauma Treatment (*n* = 58)	Did Not Receive Trauma Treatment (*n* = 46)	*t*	*df*	*p*
Age at admit (*M*, SD)		28.62 (11.29)	27.00 (10.13)	−0.77	100.41	0.442
Age at onset (*M*, SD)		12.34 (4.94)	14.18 (3.42)	2.23	98.88	**0.028**
BMI at admit (*M*, SD)		24.28 (9.20)	21.16 (3.56)	−2.37	77.06	**0.020**
BMI at discharge (*M*, SD)		26.07 (8.94)	22.53 (3.45)	−2.70	72.44	**0.009**
Years of education (*M*, SD)		14.78 (2.01)	14.34 (2.52)	−0.88	69.33	0.382
					
				$x^{2}$	*df*	*p*
Diagnosis at admit (*n*, %)				7.00	4	0.136
	Anorexia Nervosa—Restricting Type	12 (20.7%)	16 (34.8%)			
	Anorexia Nervosa—Binge/Purge Type	10 (17.2%)	10 (21.7%)			
	Bulimia Nervosa	13 (22.4%)	12 (26.1%)			
	Avoidant/Restrictive Food Intake Disorder	3 (5.2%)	0 (0.0%)			
	Other Specified Feeding and Eating Disorder	20 (34.5%)	8 (17.4%)			
Sex (*n*, %)				0.08	1	0.782
	Male	3 (5.2%)	1 (2.2%)			
	Female	55 (94.8%)	45 (97.8%)			
Gender (*n*, %)				1.43	3	0.697
	Man	3 (5.2%)	2 (4.3%)			
	Woman	51 (87.9%)	39 (84.8%)			
	Genderqueer/gender non-conforming/gender fluid	4 (6.9%)	5 (10.9%)			
Race (*n*, %)				3.27	4	0.514
	White	51 (87.9%)	40 (87%)			
	Asian	2 (3.4%)	2 (4.3%)			
	Black	0 (0.0%)	1 (2.2%)			
	Native Hawaiian/Alaska Native	1 (1.7%)	0 (0.0%)			
	Other ^a^	4 (6.9%)	1 (2.2%)			
	Did not respond	0 (0.0%)	2 (4.3%)			
Ethnicity (*n*, %)				0.45	1	0.500
	Hispanic/Latinx	13 (22.4%)	7 (15.2%)			
	Not Hispanic/Latinx	45 (77.6%)	39 (84.8%)			

Note. *M*, mean; SD, standard deviation. Bolded effects are significant at *p* < 0.05 level. ^a^ Participants identified themselves as “mixed” (*n* = 1), “Mexican” (*n* = 1), and both White and Asian (*n* = 3).

**Table 2:** Model fit results from the multilevel models.

Outcome	*n*	AIC	BIC	logLik	Marginal R^2^	Conditional R^2^	ICC
EDE-Q Dietary Restraint	104	1470.2	1501.6	−727.08	0.06	0.51	0.48
EDE-Q Eating Concern	104	1287.5	1318.9	−635.74	0.10	0.53	0.48
EDE-Q Shape Concern	104	1376.4	1407.8	−680.19	0.06	0.63	0.60
EDE-Q Weight Concern	104	1406.8	1438.3	−695.42	0.06	0.61	0.59
EDE-Q Global	104	1298.4	1329.8	−641.21	0.08	0.61	0.58
EPSI Binge Eating	93	2109.8	2140.4	−1046.92	0.03	0.71	0.70
EPSI Body Dissatisfaction	93	2082.8	2113.4	−1033.41	0.04	0.73	0.72
EPSI Cognitive Restraint	93	1635.5	1666.1	−809.76	0.06	0.76	0.74
EPSI Excessive Exercise	93	2022.7	2053.2	−1003.34	0.04	0.65	0.64
EPSI Purging	93	1827.4	1858.0	−905.72	0.03	0.66	0.65
EPSI Restricting	93	2134.1	2164.7	−1059.07	0.06	0.59	0.56
Anxiety	103	2771.2	2802.6	−1377.60	0.08	0.60	0.57
Depression	68	1589.4	1617.5	−786.68	0.08	0.52	0.48
Impairment	93	2583.3	2613.9	−1283.67	0.14	0.57	0.50

**Table 3:** Fixed effects results from the multilevel models.

Outcome	Fixed Effect	Estimate	SE	*t*	*df*	*p*
EDE-Q Dietary Restraint						
	Intercept	2.93	0.25	11.64	98.37	**<0.001**
	Time	−0.30	0.09	−3.26	77.63	**0.002**
	Trauma Treatment	0.06	0.33	0.19	96.56	0.849
	Trauma Treatment × Time	−0.06	0.12	−0.47	73.54	0.639
EDE-Q Eating Concern						
	Intercept	3.23	0.19	17.23	95.72	**<0.001**
	Time	−0.28	0.07	−4.13	75.57	**<0.001**
	Trauma Treatment	−0.04	0.25	−0.18	93.84	0.860
	Trauma Treatment × Time	−0.11	0.09	−1.22	71.49	0.227
EDE-Q Shape Concern						
	Intercept	4.66	0.22	21.48	100.66	**<0.001**
	Time	−0.26	0.08	−3.34	73.54	**0.001**
	Trauma Treatment	0.17	0.29	0.58	98.73	0.562
	Trauma Treatment × Time	−0.13	0.10	−1.26	69.54	0.213
EDE-Q Weight Concern						
	Intercept	4.19	0.23	18.57	101.16	**<0.001**
	Time	−0.28	0.08	−3.33	70.02	**0.001**
	Trauma Treatment	0.25	0.30	0.83	99.23	0.407
	Trauma Treatment × Time	−0.09	0.11	−0.86	66.02	0.395
EDE-Q Global						
	Intercept	3.75	0.20	18.91	97.98	**<0.001**
	Time	−0.28	0.07	−3.94	73.48	**<0.001**
	Trauma Treatment	0.12	0.26	0.45	96.14	0.654
	Trauma Treatment × Time	−0.10	0.09	−1.03	69.44	0.308
EPSI Binge Eating						
	Intercept	9.87	1.25	7.91	91.50	**<0.001**
	Time	−0.30	0.29	−1.03	68.25	0.308
	Trauma Treatment	−0.99	1.65	−0.60	89.07	0.550
	Trauma Treatment × Time	−0.56	0.37	−1.49	64.74	0.142
EPSI Body Dissatisfaction						
	Intercept	19.81	0.96	20.68	90.12	**<0.001**
	Time	−0.29	0.31	−0.94	76.19	0.349
	Trauma Treatment	2.68	1.26	2.13	87.72	**0.036**
	Trauma Treatment × Time	−0.93	0.40	−2.29	71.52	**0.025**
EPSI Cognitive Restraint						
	Intercept	7.29	0.54	13.55	87.96	**<0.001**
	Time	−0.44	0.17	−2.58	81.82	0.012
	Trauma Treatment	0.63	0.71	0.89	85.82	0.376
	Trauma Treatment × Time	−0.31	0.22	−1.38	77.44	0.173
EPSI Excessive Exercise						
	Intercept	6.61	1.04	6.33	85.16	**<0.001**
	Time	−0.43	0.32	−1.34	64.23	0.186
	Trauma Treatment	1.76	1.37	1.29	83.83	0.202
	Trauma Treatment × Time	−0.43	0.42	−1.03	60.89	0.307
EPSI Purging						
	Intercept	4.71	0.75	6.26	90.91	**<0.001**
	Time	−0.37	0.21	−1.73	87.04	0.087
	Trauma Treatment	−0.12	0.99	−0.13	88.80	0.900
	Trauma Treatment × Time	−0.27	0.28	−0.98	81.89	0.332
EPSI Restricting						
	Intercept	14.17	1.02	13.84	87.57	**<0.001**
	Time	−1.11	0.35	−3.15	75.52	**0.002**
	Trauma Treatment	0.12	1.34	0.09	86.02	0.931
	Trauma Treatment × Time	−0.19	0.46	−0.41	71.05	0.686
Anxiety						
	Intercept	65.24	1.32	49.42	99.72	**<0.001**
	Time	−2.28	0.57	−4.02	88.84	**<0.001**
	Trauma Treatment	0.76	1.75	0.43	96.91	0.667
	Trauma Treatment × Time	−0.24	0.75	−0.33	83.73	0.744
Depression	Intercept	17.76	1.02	17.42	61.19	**<0.001**
	Time	−0.93	0.42	−2.23	54.93	**0.030**
	Trauma Treatment	−0.27	1.33	−0.21	60.20	0.838
	Trauma Treatment × Time	−0.49	0.54	−0.91	53.22	0.369
Clinical Impairment						
	Intercept	31.84	1.70	18.75	83.05	**<0.001**
	Time	−3.09	0.64	−4.87	80.70	**<0.001**
	Trauma Treatment	2.95	2.22	1.33	81.09	0.188
	Trauma Treatment × Time	−0.89	0.83	−1.07	75.43	0.288

Note. SE, standard error. Bolded effects are significant at *p* < 0.05 level.

## Data Availability

The study’s data are not currently publicly available, but may be shared upon reasonable request.

## Supplementary Materials

The following supporting information can be downloaded at: https://www.mdpi.com/article/10.3390/bs15091173/s1, Table S1: Post hoc power estimates for fixed effects by model.

## Abbreviations

The abbreviations below are used in this manuscript.

PTSD	Posttraumatic Stress Disorder
EDs	Eating Disorders
ED-PTSD	Co-occurring Eating Disorders and Posttraumatic Stress Disorder
AN	Anorexia Nervosa
BN	Bulimia Nervosa
PE	Prolonged Exposure
CPT	Cognitive Processing Therapy
CBT	Cognitive Behavioral Therapy
TAU	Treatment as Usual
EDE-Q	Eating Disorder Examination Questionnaire

## References

[B1-behavsci-15-01173] Arend M. G., Schäfer T. (2019). Statistical power in two-level models: A tutorial based on monte carlo simulation. Psychological Methods.

[B2-behavsci-15-01173] Bates D., Mächler M., Bolker B., Walker S. (2015). Fitting linear mixed-effects models using lme4. Journal of Statistical Software.

[B3-behavsci-15-01173] Bohn K., Fairburn C. G., Fairburn C. G. (2008). Clinical impairment assessment questionnaire (CIA 3.0). Cognitive behavior therapy and eating disorders.

[B4-behavsci-15-01173] Brewerton T. D. (2019). An overview of trauma-informed care and practice for eating disorders. Journal of Aggression, Maltreatment, & Trauma.

[B5-behavsci-15-01173] Brewerton T. D. (2023). The integrated treatment of eating disorders, posttraumatic stress disorder, and psychiatric comorbidity: A commentary on the evolution of principles and guidelines. Frontiers in Psychiatry.

[B6-behavsci-15-01173] Brown T. A., Cusack A., Anderson L. K., Trim J., Nakamura T., Trunko M. E., Kaye W. H. (2018). Efficacy of a partial hospital programme for adults with eating disorders. European Eating Disorders Review.

[B7-behavsci-15-01173] Carbajal J. (2018). Trauma-focused interventions: A clinical practice analysis. The Practitioner Scholar: Journal of Counseling and Professional Psychology.

[B8-behavsci-15-01173] Claudat K., Reilly E. E., Convertino A. D., Trim J., Cusack A., Kaye W. H. (2022). Integrating evidence-based PTSD treatment into intensive eating disorders treatment: A preliminary investigation. Eating and Weight Disorders.

[B9-behavsci-15-01173] Convertino A. D., Mendoza R. R. (2023). Posttraumatic stress disorder, traumatic events, and longitudinal eating disorder treatment outcomes: A systematic review. The International Journal of Eating Disorders.

[B10-behavsci-15-01173] Ennis N., Shorer S., Shoval-Zuckerman Y., Freedman S., Monson C. M., Dekel R. (2020). Treating posttraumatic stress disorder across cultures: A systematic review of cultural adaptations of trauma-focused cognitive behavioral therapies. Journal of Clinical Psychology.

[B11-behavsci-15-01173] Fairburn C. G., Beglin S. J. (1994). Assessment of eating disorders: Interview or self-report questionnaire?. The International Journal of Eating Disorders.

[B12-behavsci-15-01173] Ferrell E. L., Russin S. E., Flint D. D. (2022). Prevalence estimates of comorbid eating disorders and posttraumatic stress disorder: A quantitative synthesis. Journal of Aggression, Maltreatment & Trauma.

[B13-behavsci-15-01173] First M. B., Williams J. B., Karg R. S., Spitzer R. L. (2015). Structured clinical interview for DSM-5—Research version (SCID-5 for DSM-5, research version; SCID-5-RV).

[B14-behavsci-15-01173] Foa E. B., Hembree E. A., Rothbaum B. O., Rauch S. A. M. (2019). Prolonged exposure therapy for PTSD: Emotional processing of traumatic experiences—Therapist guide.

[B15-behavsci-15-01173] Forbush K. T., Wildes J. E., Pollack L. O., Dunbar D., Luo J., Patterson K., Petruzzi L., Pollpeter M., Miller H., Stone A., Bright A., Watson D. (2013). Development and validation of the Eating Pathology Symptoms Inventory (EPSI). Psychological Assessment.

[B16-behavsci-15-01173] Glashouwer K. A., van der Veer R. M. L., Adipatria F., de Jong P. J., Vocks S. (2019). The role of body image disturbance in the onset, maintenance, and relapse of anorexia nervosa: A systematic review. Clinical Psychology Review.

[B17-behavsci-15-01173] Gleaves D. H., Eberenz K. P., May M. C. (1998). Scope and significance of posttraumatic symptomatology among women hospitalized for an eating disorder. The International Journal of Eating Disorders.

[B18-behavsci-15-01173] Green P., MacLeod C. J. (2016). SIMR: An R package for power analysis of generalized linear mixed models by simulation. Methods in Ecology and Evolution.

[B19-behavsci-15-01173] Harned M. S., Korslund K. E., Foa E. B., Linehan M. M. (2012). Treating PTSD in suicidal and self-injuring women with borderline personality disorder: Development and preliminary evaluation of a dialectical behavior therapy prolonged exposure protocol. Behaviour Research and Therapy.

[B20-behavsci-15-01173] Johnson J. G., Spitzer R. L., Williams J. B. W. (2001). Health problems, impairment and illnesses associated with bulimia nervosa and binge eating disorder among primary care and obstetric gynaecology patients. Psychological Medicine.

[B21-behavsci-15-01173] Kroenke K., Spitzer R. L. (2002). The PHQ-9: A New depression diagnostic and severity measure. Psychiatric Annals.

[B22-behavsci-15-01173] Linardon J., Wade T. D., de la Piedad Garcia X., Brennan L. (2017). The efficacy of cognitive-behavioral therapy for eating disorders: A systematic review and meta-analysis. Journal of Consulting and Clinical Psychology.

[B23-behavsci-15-01173] Mitchell K. S., Scioli E. R., Galovski T., Belfer P. L., Cooper Z. (2021). Posttraumatic stress disorder and eating disorders: Maintaining mechanisms and treatment targets. Eating Disorders.

[B24-behavsci-15-01173] R Core Team (2025). R: A language and environment for statistical computing (Version 4.5.0) *[Computer Software]*.

[B25-behavsci-15-01173] Resick P. A., Monson C. M., Chard K. M. (2017). Cognitive processing therapy for PTSD: A comprehensive manual.

[B26-behavsci-15-01173] Rienecke R. D., Blalock D. V., Mills H. D., Duffy A., Manwaring J., Le Grange D., Mehler P. S., McClanahan S., Johnson C. (2022). Treatment outcome for adults in a residential program for binge eating spectrum disorders: Protocol for a prospective pragmatic single-arm trial. JMIR Research Protocols.

[B27-behavsci-15-01173] Sheehan D. V., Lecrubier Y., Sheehan K. H., Amorim P., Janavs J., Weiller E., Hergueta T., Baker R., Dunbar G. C. (1998). The mini-international neuropsychiatric interview (M.I.N.I.): The development and validation of a structured diagnostic psychiatric interview for DSM-IV and ICD-10. The Journal of Clinical Psychiatry.

[B28-behavsci-15-01173] Speilberger C. D., Gorsuch R., Lushene R., Vagg P., Jacobs G. (1983). Manual for the state-trait anxiety inventory (form Y1–Y2).

[B29-behavsci-15-01173] Stice E., Shaw H. E. (2002). Role of body dissatisfaction in the onset and maintenance of eating pathology: A synthesis of research findings. Journal of Psychosomatic Research.

[B30-behavsci-15-01173] Tan E. J., Raut T., Le L. K.-D., Hay P., Ananthapavan J., Lee Y. Y., Mihalopoulos C. (2023). The association between eating disorders and mental health: An umbrella review. Journal of Eating Disorders.

[B31-behavsci-15-01173] Trottier K., Monson C. M., Wonderlich S. A., Crosby R. D. (2022). Results of the first randomized controlled trial of integrated cognitive-behavioral therapy for eating disorders and posttraumatic stress disorder. Psychological Medicine.

[B32-behavsci-15-01173] Trottier K., Monson C. M., Wonderlich S. A., Olmsted M. P. (2017). Initial findings from Project Recover: Overcoming co-occurring eating disorders and posttraumatic stress disorder through integrated treatment. Journal of Traumatic Stress.

[B33-behavsci-15-01173] Trottier K., Wonderlich S. A., Monson C. M., Crosby R. D., Olmsted M. P. (2016). Investigating posttraumatic stress disorder as a psychological maintaining factor of eating disorders. The International Journal of Eating Disorders.

[B34-behavsci-15-01173] Vanzhula I. A., Calebs B., Fewell L., Levinson C. A. (2019). Illness pathways between eating disorder and post-traumatic stress disorder symptoms: Understanding comorbidity with network analysis. European Eating Disorders Review.

