# Supplementary material for: Exploring the Additive Benefit of PTSD Treatment on Eating Disorder Outcomes for Those with Co-Occurring PTSD
Source: Behav Sci (Basel). 2025 Aug 29;15(9):1173. doi: 10.3390/bs15091173 (PMC12466857; doi:10.3390/bs15091173)
Supplement: Supplementary file 1 [file behavsci-15-01173-s001.zip › behavsci-3704090-supplementary.pdf]

Table S1. Post hoc power estimates for fixed effects by model.

Model	Fixed Effect	Estimated Power	95% CI
EDE-Q Dietary Restraint			
	Time	0.993	[0.986, 0.997]
	Trauma Treatment	0.388	[0.358, 0.419]
	Trauma Treatment × Time	0.905	[0.885, 0.922]
EDE-Q Eating Concern			
	Time	0.997	[0.991, 0.999]
	Trauma Treatment	0.449	[0.418, 0.480]
	Trauma Treatment × Time	0.957	[0.943, 0.969]
EDE-Q Shape Concern			
	Time	1.000	[0.996, 1.000]
	Trauma Treatment	0.468	[0.437, 0.499]
	Trauma Treatment × Time	0.960	[0.946, 0.971]
EDE-Q Weight Concern			
	Time	0.998	[0.993, 1.000]
	Trauma Treatment	0.470	[0.439, 0.501]
	Trauma Treatment × Time	0.952	[0.937, 0.964]
EDE-Q Global			
	Time	0.998	[0.993, 1.000]
	Trauma Treatment	0.479	[0.448, 0.510]
	Trauma Treatment × Time	0.954	[0.939, 0.966]
EPSI Binge Eating			
	Time	1.000	[0.996, 1.000]
	Trauma Treatment	0.250	[0.223, 0.278]
	Trauma Treatment × Time	0.975	[0.963, 0.984]
EPSI Body Dissatisfaction			
	Time	0.997	[0.991, 0.999]
	Trauma Treatment	0.374	[0.344, 0.405]
	Trauma Treatment × Time	0.952	[0.937, 0.964]
EPSI Cognitive Restraint			
	Time	0.990	[0.982, 0.995]
	Trauma Treatment	0.328	[0.299, 0.358]
	Trauma Treatment × Time	0.925	[0.907, 0.941]
EPSI Excessive Exercise			
	Time	0.964	[0.951, 0.975]
	Trauma Treatment	0.238	[0.212, 0.266]
	Trauma Treatment × Time	0.840	[0.816, 0.862]
EPSI Purging			

	Time	0.996	[0.990, 0.999]
	Trauma Treatment	0.267	[0.240, 0.296]
	Trauma Treatment × Time	0.921	[0.903, 0.937]
EPSI Restricting			
	Time	0.977	[0.966, 0.985]
	Trauma Treatment	0.312	[0.283, 0.342]
	Trauma Treatment × Time	0.867	[0.844, 0.887]
Anxiety			
	Time	0.993	[0.986, 0.997]
	Trauma Treatment	0.528	[0.497, 0.559]
	Trauma Treatment × Time	0.936	[0.919, 0.950]
Depression			
	Time	0.896	[0.875, 0.914]
	Trauma Treatment	0.323	[0.294, 0.353]
	Trauma Treatment × Time	0.706	[0.677, 0.734]
Clinical Impairment			
	Time	0.991	[0.983, 0.996]
	Trauma Treatment	0.408	[0.377, 0.439]
	Trauma Treatment × Time	0.930	[0.912, 0.945]

Note. CI, confidence interval. Results are based on 1,000 Monte Carlo simulations and assume a standardized medium effect size (Cohen's *d* = 0.5) for all fixed effects.
